# Supplementary material for: RASSF1A Suppression as a Potential Regulator of Mechano-Pathobiology Associated with Mammographic Density in BRCA Mutation Carriers
Source: Cancers (Basel). 2021 Jun 29;13(13):3251. doi: 10.3390/cancers13133251 (PMC8269117; doi:10.3390/cancers13133251)
Supplement: Supplementary file 1 [file cancers-13-03251-s001.zip › cancers-1223847-supplementary.pdf]

Supplementary Figure S1

Reye et al., 2021

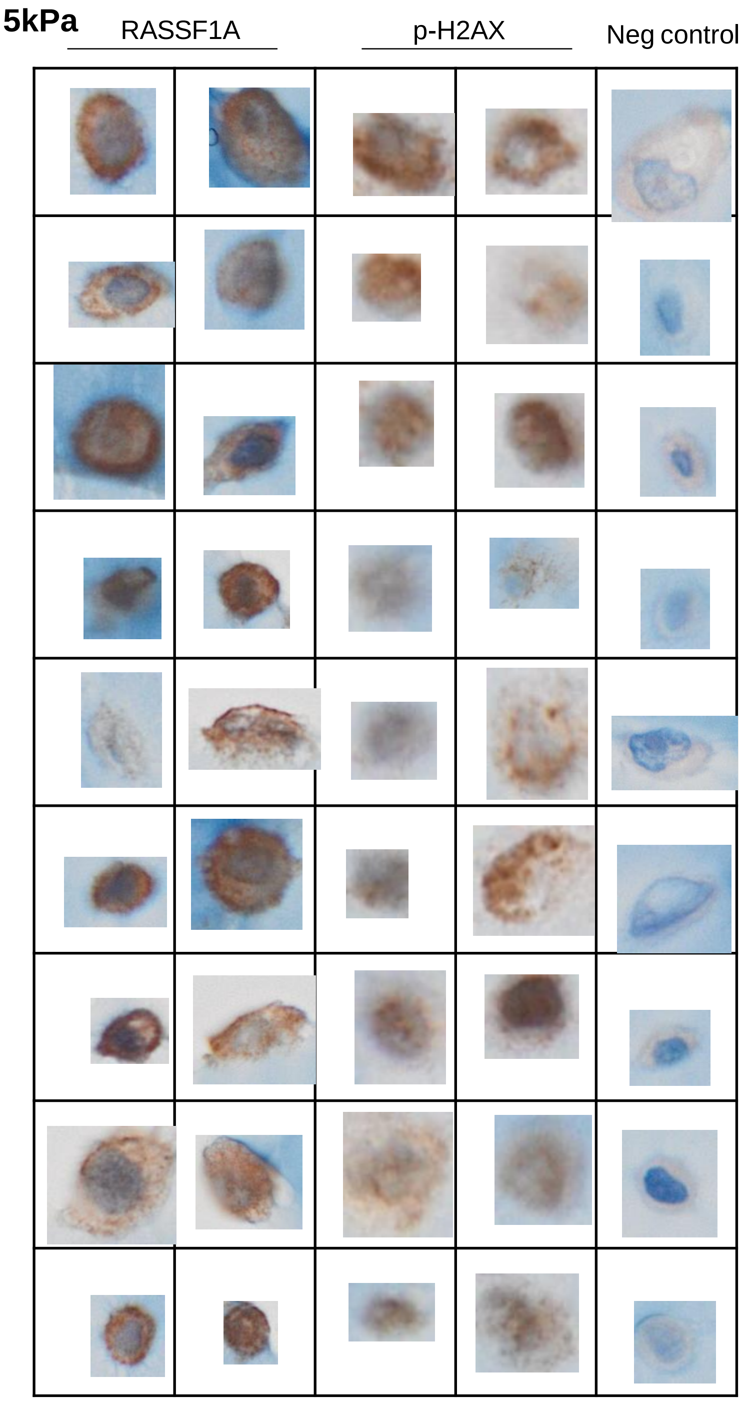

Supplementary Figure S2

Reye et al., 2021

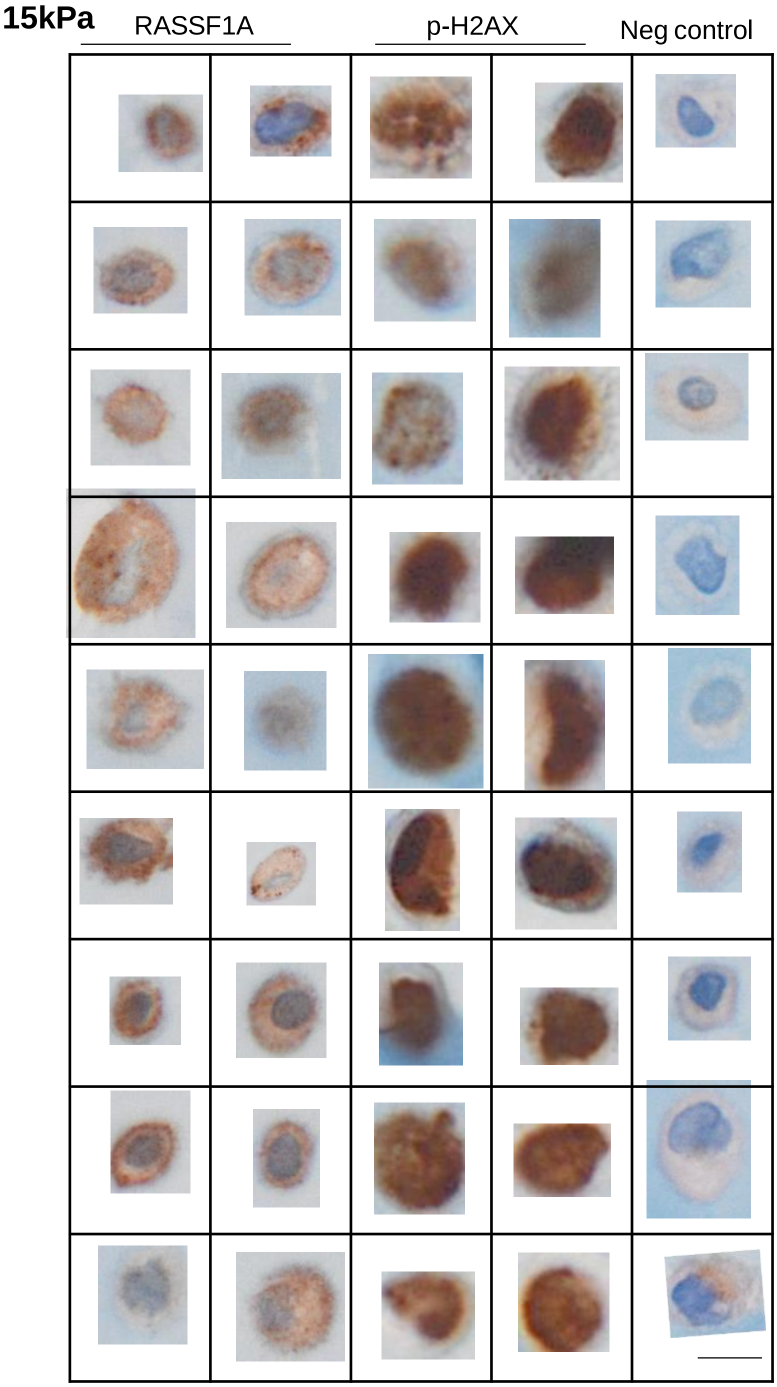

Supplementary Figure S3

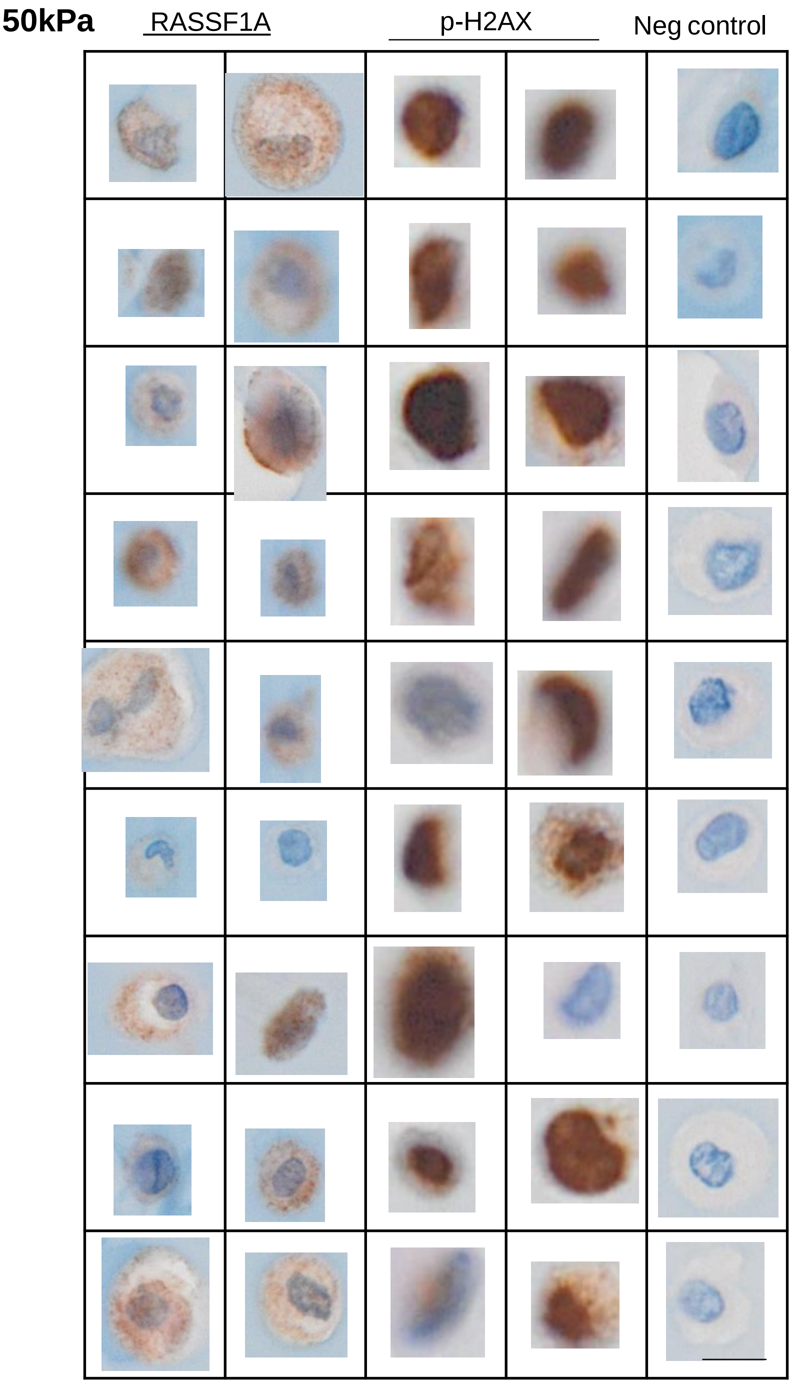

Supplementary Figure S4

Stiffness (kPa)	Activated caspase-3
5	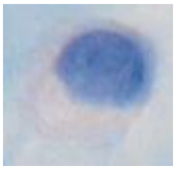
15	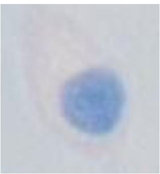
50	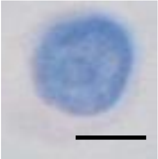

Supplementary Figure S5

Reye et al., 2021

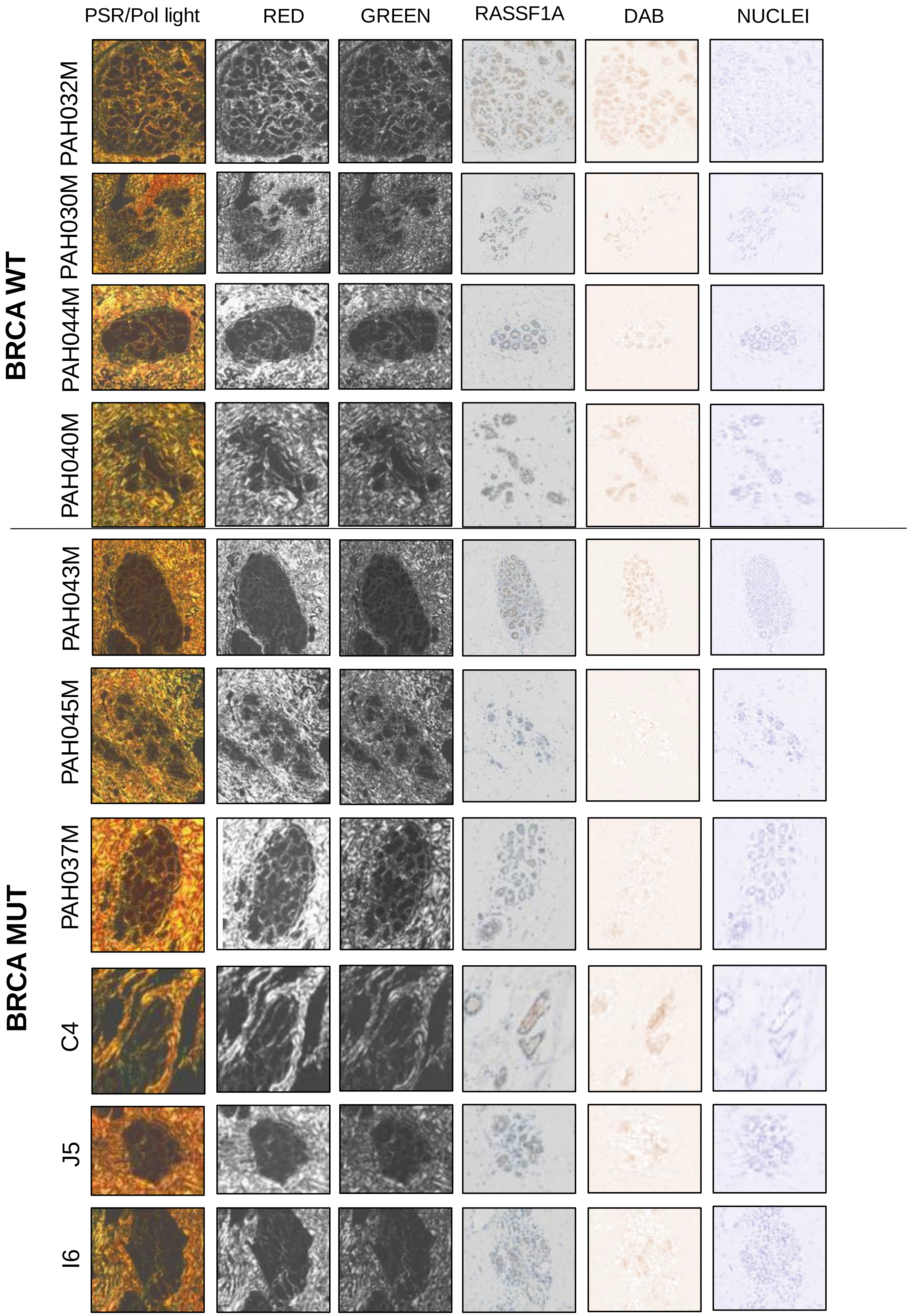

Supplementary Figure S6

A

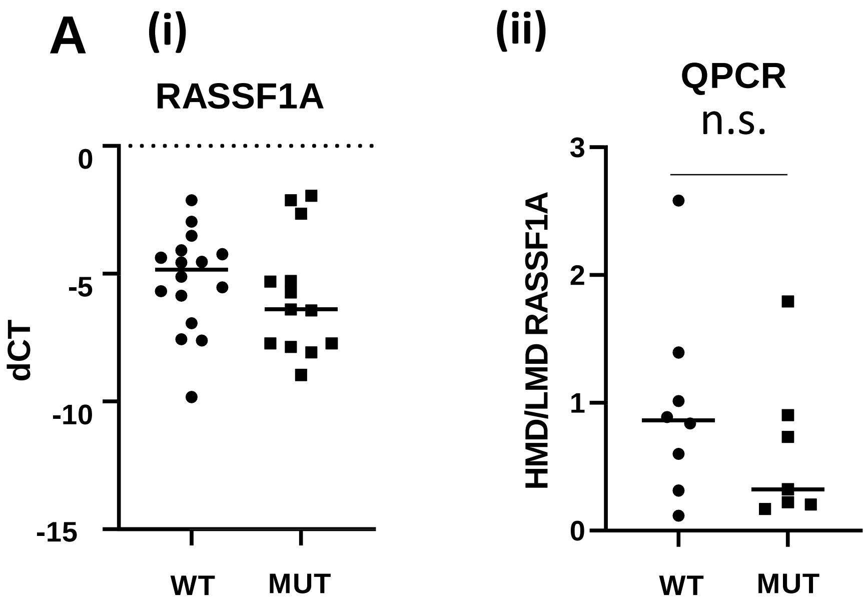

Supplementary Table S1

Risk factor	Population 10-year risk (%)
BiRADs cat 1 (LMD)	1.0
BiRADs cat 4 (HMD)	3.6
BRCA1 mutation + BiRADs cat 1 (LMD)	13.3
BRCA2 mutation + BiRADs cat 1 (LMD)	7.9
BRCA1 mutation + BiRADs cat 4 (HMD)	38.20
BRCA2 mutation + BiRADs cat 4 (HMD)	24.90
